# Elucidating vaginal fistulas on CT and MRI

**DOI:** 10.1186/s13244-019-0812-9

**Published:** 2019-12-18

**Authors:** Massimo Tonolini

**Affiliations:** Department of Radiology, “Luigi Sacco” University Hospital, Via G.B. Grassi 74, 20157 Milan, Italy

**Keywords:** Vagina, Fistula, Iatrogenic injury, Computed tomography, Magnetic resonance imaging

## Abstract

Vaginal fistulas (VF) represent abnormal communications between the vagina and either the distal portion of the digestive system or the lower urinary tract, but lack an accepted classification and standardised terminology. Regardless of the underlying cause, these uncommon disorders result in profound physical, psychological, sexual and social distress to the patients.

Since diagnosis of VF is challenging at gynaecologic examination, ano-proctoscopy and urethro-cystoscopy, imaging is crucial to confirm the fistula, to visualise its site, course and involved organ, and to characterise the underlying disease. The traditional conventional radiographic studies provided limited cross-sectional information and are nowadays largely replaced by CT and MRI studies.

Aiming to provide radiologists with an increased familiarity with VF, this pictorial paper summarises their clinical features, pathogenesis and therapeutic approach, and presents the appropriate CT and MRI acquisition and interpretation techniques that vary according to the anatomic site and termination of the fistula. The current role of state-of-the art CT and MRI is presented with examples regarding both entero- (involving the colon, rectum and anus) and urinary (connecting the bladder, distal ureter or urethra) VF. The resulting combined anatomic and functional cross-sectional information is crucial to allow a correct therapeutic choice and surgical planning.

## Key points

- Vaginal fistulas are broadly categorised as they affect either the distal bowel (sigmoid colon, rectus, anal canal) or urinary tract (bladder, distal ureter or urethra)
- Among the spectrum of causes, in Western countries, iatrogenic surgical injuries represent an increasing concern
- CT diagnosis of bowel vaginal fistulas benefits from small field-of-view oblique and sagittal interpretation, and optional intrarectal contrast
- If not contraindicated, focused MRI provides superior visualisation of ano- and rectovaginal fistulas
- CT-urography and additional CT-cystography now represent the mainstay techniques to diagnose urinary VF

## Introduction

Fistulas of the female genital tract were described in medical literature since ancient times: among them, vaginal fistulas (VF) are the most prevalent and are defined as abnormal epithelium-lined communications between the vagina and other pelvic organs [1]. The spectrum of causes encompasses congenital and developmental abnormalities, inflammatory diseases, infections, tumours, sexual and obstetric trauma, irradiation and post-surgical injuries [2]. Regardless of the underlying disorder, all VF result in substantial morbidity and severely impair the patients’ quality of life [3–5].

The majority of patients with VF are initially referred to a gynaecologist; however, despite more or less evident clinical signs, vaginal exploration may identify the fistulous orifice in less than 80% of cases [5]. Similarly, visualisation of the abnormal communication is generally quite challenging at either ano-proctoscopy or urethro-cystoscopy. As a result, clinicians and surgeons need critical help from radiologists to (1) confirm the presence of a VF, (2) visualise its site, course and involved organs, and (3) characterise the underlying pathology [6, 7].

Traditionally, imaging demonstration of VF relied on fluoroscopic studies such as contrast medium (CM) enema, intravenous excretory urography, voiding and retrograde cystography, which may opacify a patent fistulous tract but provide very limited information on the affected organs [8, 9]. Although developed as the best radiographic technique to confirm and visualise a VF by pressure, conventional vaginography is relatively invasive, cumbersome and poorly tolerated as it requires obstruction of the vaginal introitus by an inflated Foley catheter before injection of CM [9].

In recent years, CT and MRI studies are largely replacing conventional radiologic techniques. With appropriate acquisition and focused interpretation, state-of-the art cross-sectional imaging may provide optimal visualisation of VF, involved organs and underlying diseases, which is crucial for correct choice between conservative and surgical treatment and appropriate surgical planning. Aiming to improve radiologists’ familiarity with these uncommon but challenging entities, this pictorial essay provides a concise review of VF types, clinical features, causes and mechanisms, then presents with examples the state-of-the art CT and MRI techniques and appearances of VF.

## Clinical overview of vaginal fistulas

### Types and causes

Although lacking an accepted classification scheme or standardised terminology, VF may be broadly separated into either entero- or urinary VF according to involvement of the distal bowel or lower urinary tract, respectively. Both categories are further subdivided on the basis of the target organ [2, 10].

**Fig. 1:**
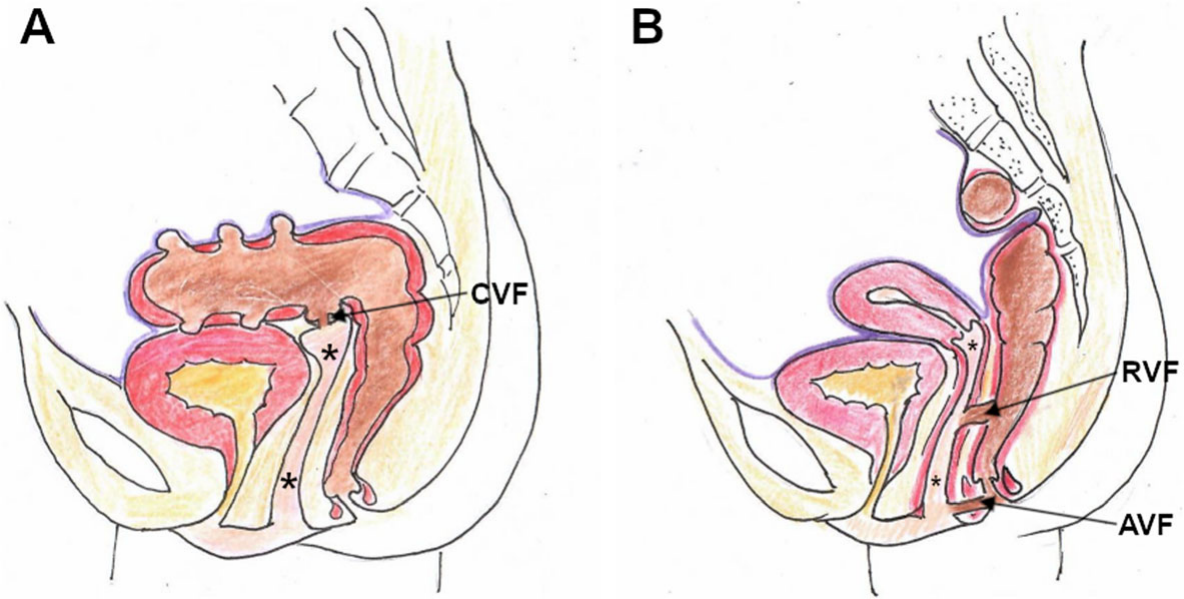
Schematic representation of entero-vaginal fistulas (VF). **a** colo-VF (CVF). **b** recto-VF (RVF) and ano-VF (AVF). Note absent uterus in (**a**). Vagina indicated by asterisk (*), urine in yellow, stools in brown

Entero-VF (Fig. 1) may involve the sigmoid colon (colo-VF), rectum (recto-VF) or anus (ano-VF). Their underlying causes and mechanisms are summarised in Table 1 [1, 5, 11].

**Table 1:** Categorisation, causes and mechanisms of entero-vaginal fistulas

Type	Cause	Notes
Colovaginal	Complicated colonic diverticulitis	Patients with prior hysterectomy Either (a) inflamed sigmoid colon directly adheres to the vaginal vault or (b) via formation of interposed abscess that opens in the vagina
Rectovaginal	Past irradiation such as for uterine cervix carcinoma	Delayed onset (years after treatment) Increasingly uncommon
	Primary or recurrent pelvic tumours	Either (a) rectal carcinoma invading the vagina or (b) gynaecologic malignancies invading the rectum
	Surgical injury	
	- Low anterior resection for rectal cancer	Risk up to 5–10% of patients, part of anastomotic leakage spectrum Inadvertent clipping of vagina in staples
	- Pelvic floor surgery	With positioning of prosthetic mesh
	- Haemorrhoid surgery	
Anovaginal	Crohn’s disease (CD)	CD = 25% of all vaginal fistulas (VF) VF < 4–9% of all CD-related perianal inflammatory disease Often complex forms
	Ulcerative colitis	Perianal inflammatory disease (rare) Ileal pouch-anal anastomosis leakage
	Cryptoglandular or other inflammation	E.g. Bartholin’s gland abscess
	Perineal laceration	From either (a) direct trauma (often sexual violence) or (b) obstetric injury (spontaneous or instrumental delivery)

**Fig. 2:**
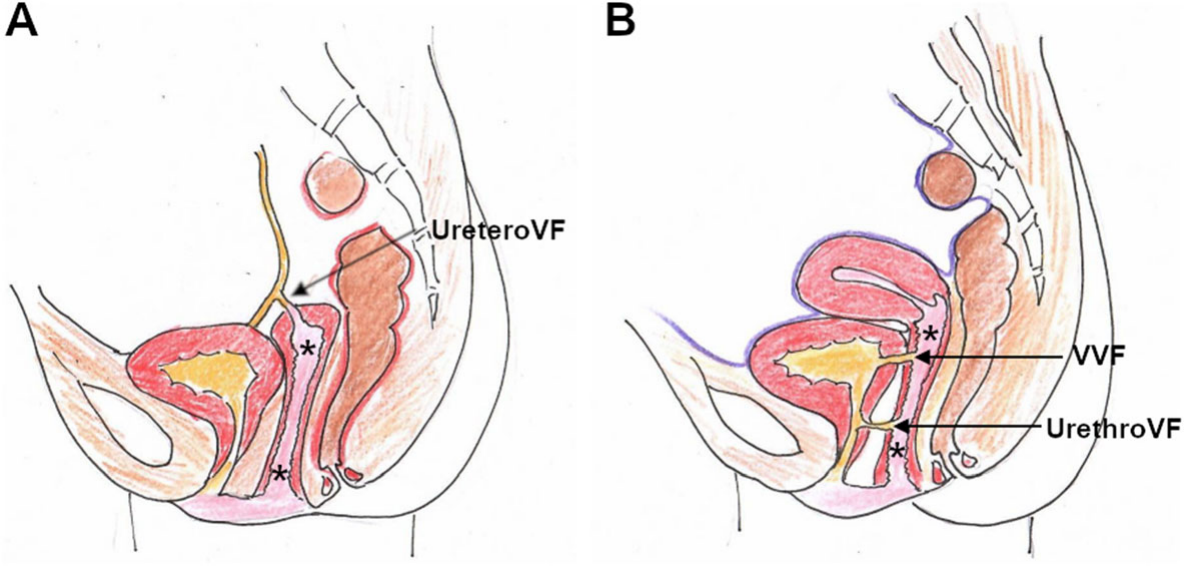
Schematic representation of urinary VF. **a** uretero-VF (CVF). **b** vesico-VF (VVF) and urethro-VF. Note absent uterus in (**a**). Vagina indicated by asterisk (*), urine in yellow, stools in brown

In developing countries, VF are still common and almost invariably secondary to obstructed labour [4, 12]. Conversely, despite advancements in open and laparoscopic surgical techniques, in the Western world, over 90% of all urinary VF (Fig. 2, Table 2) now develop as iatrogenic complications of irradiation or surgical injury to either distal ureter or bladder. However, a post-surgical VF is rather uncommon (2% of cases) compared with bladder (60–70%) and ureteral (24–30% of cases) injuries without vaginal involvement [13–16].

**Table 2:** Categorisation, causes and mechanisms of urinary vaginal fistulas

Type	Cause	Notes
Ureterovaginal	Surgical injury	Intraoperative injury to the distal ureter Risk further increased by parametrial and nodal dissection Often via formation of urinoma that drains into the vaginal vault
	- Most frequent (75% of cases): total abdominal or radical hysterectomy	
	- Less common procedures: laparoscopic treatment of endometriosis, surgery for ovarian cancer, complex urological or lower gastrointestinal pelvic surgeries	
Vesicovaginal + urethrovaginal	Surgical injury	Intraoperative injury to urinary bladder Often with formation of urinoma that drains into the vaginal vault Sometimes via necrosis of vaginal vault from incorrectly placed sutures between the vaginal cuff and posterior aspect of bladder
	- Same interventions as above plus	
	- Emergency caesarean section	
	- Anti-incontinence procedures, cystocele repair, resection of urethral diverticulum	
	Locally advanced malignancies	Rare, e.g. uterine cervix carcinomas, urethral/bladder transitional carcinomas
	Past irradiation such as for uterine cervix carcinoma	Delayed onset (years after treatment) Increasingly uncommon
	Perineal laceration	From direct trauma (most usually sexual violence)
	Obstetric complication (spontaneous or instrumental delivery)	Historical, still today in developing countries lacking obstetric practices Via pressure necrosis of the anterior vaginal wall and bladder neck, compressed between the foetal head and the symphysis pubis

### Manifestations

Regardless of type, all VF cause distressing symptoms including persistent vaginitis despite treatment, dyspareunia, painful perineal dermatitis and excoriation. Entero-VF are heralded by foul-smelling enteral or faecal discharge through the vagina. Faecal incontinence may develop secondary to associated loss of anal sphincter function [1, 5].

The characteristic symptom of urinary VF is continual leakage of urine from the vagina and vulvar irritation. In recently operated patients, specific symptoms of VF are often masked by common post-operative problems such as abdominal and flank pain, hematuria, worsening renal function, fever and paralytic ileus. Not unusually, iatrogenic damage to the urinary tract is heralded by imaging detection of fluid collections representing urinoma. Biochemical assay of discharge fluid for creatinine levels and intravesical injection of methylene blue dye are helpful to confirm the presence of the VF [3, 4].

## Cross-sectional imaging techniques

### MRI indications and protocol

MRI arguably represents the best imaging modality to visualise the normal pelvic and perineal structures including the anal sphincter muscles and to elucidate suspected vaginal disorders [17, 18]. The strength of MRI relies on its ability to identify acute inflammatory changes and abscesses, post-surgical fibrosis and neoplastic tissue. Therefore, if allowed by the patient’s clinical conditions, MRI is the preferred imaging modality to investigate suspected urethro-, vesico-, ano- and recto-VF [2, 6].

In particular, the most recent European Crohn’s and Colitis Organisation (ECCO) guidelines recommend MRI as the first-line, most accurate modality for preoperative diagnosis of perianal involvement in Crohn’s disease (CD), as it dramatically affects medical and surgical therapy planning and improves the outcome [19].

Apart from fasting some hours before the examination, generally no special bowel preparation is required before MRI. However, some centres suggest that preliminary distension with ultrasound gel may ease identification of the vagina [6]. Ideally, the urinary bladder should be moderately distended. Patients are scanned in the supine position, with a phased-array coil placed at the height of the pubic symphysis [6, 17, 18].

The MRI acquisition protocol adopted on our clinical 1.5 T MRI scanner (Ingenia, Philips—the Netherlands) is presented in Table 3 and mostly relies on multiplanar T2-weighted images with a limited field-of-view (FOV). In the setting of known or suspected perianal inflammatory disease, the axial and coronal images should be oriented along oblique planes, respectively perpendicular and parallel to the main axis of the anal canal identified on the midline sagittal image. The routine inclusion of fat-suppressed (FS) heavily T2-weighted acquisitions improves detection of fluid-containing fistulas or abscesses and of inflammatory changes of the vaginal wall and perivisceral fat [20, 21]. Additionally, perianal fistulas and abscesses have higher conspicuity against the suppressed background signal on high *b* value diffusion-weighted (DW) acquisition compared with T2-weighted images [20, 22].

**Table 3:** Focused MRI acquisition protocol for the study of the ano-perineal region

Sequence	Orientation	FOV (mm)	Matrix	No. of slices	Section thickness (mm)^b^	No. of averages	TR/TE (ms) (range)^c^
T2-w TSE	Sagittal	240	344 × 388	25	3.5	1–2	2500–5000/90
T2-w TSE	Axial/oblique-axial	220	320 × 311	35–40	3	2	2800–5000/90
T2-w TSE	Coronal/oblique-coronal	240	344 × 388	25	3.5	1–2	2500–5000/90
FS T2-w SPAIR	Axial	240	268 × 262	30–35	3.5	2	3000–5000/90
EPI DW	Axial	240	75 × 65	30–35	3.5	3	Shortest^d^
(if not contraindicated) i.v. paramagnetic contrast medium such as as 1-M gadobutrol (Gadovist, Bayer – Germany) 0.1 ml/kg							
FS T1-w SPIR^a^	Sagittal	240	344 × 388	25	3	1–2	400–700/10
FS T1-w SPIR^a^	Axial/oblique-axial	220	320 × 311	35–40	3.5	2	400–700/10
FS T1-w SPIR^a^ (optional)	Coronal/oblique-coronal	240	344 × 388	25	3.5	1–2	400–700/10

*FOV* field-of-view, *TR* repetition time, *TE* echo time, *T2-w* T2-weighted, *T1-w* T1-weighted, *TSE* turbo spin-echo, *SPAIR* Spectral Attenuated Inversion Recovery, *EPI* echo-planar imaging, *DW* diffusion-weighted imaging, *FS* fat-suppressed, *SPIR* Spectral Presaturation with Inversion Recovery - ^a^may be replaced with volumetric FS T1-w gradient-echo sequences such as THRIVE (T1 high-resolution isotropic volume excitation), LAVA (liver acquisition with volume acquisition) or VIBE (volumetric interpolated breath-hold examination). ^b^Minimal (10%) intersection gap. ^c^Automatically selected in relation to geometrical parameters. ^d^Four *b* values (0… 1000 mm^2^/s)

Non-contrast MRI sequences may be sufficient to visualise simple ano- and urethro-VF. However, intravenous CM may be helpful to elucidate fistulas with obliterated walls, which are usually difficult to identify on precontrast images. If not contraindicated by allergy or impaired renal function, studies are generally completed with multiplanar FS T1-weighted images after intravenous gadolinium CM that allow detection of enhancement in active, inflamed VF and abscess walls. A thorough CM-enhanced MRI is mandatory in patients with known or suspected CD and neoplastic disease [6, 20, 21].

### CT indications and techniques for the lower digestive tract

Albeit with a lower contrast resolution compared with MRI, many patients with entero-VF are initially investigated with multidetector CT. According to our experience, the use of CT should be reserved: (1) as a first-line investigation in emergency department patients with acute abdominal complaints, such as acute diverticulitis for which CT represents the mainstay technique for diagnosis and staging, (2) when MRI is unavailable, intolerable or contraindicated by metallic foreign bodies, pacemaker or other MRI-unsafe device, (3) in elderly or critically ill women, and (4) in the early post-operative setting and other questionable cases where further investigation with intraluminal CM is considered [2, 6, 7].

CT studies should include at least a portal-venous phase of enhancement after intravenous injection of 110–130 mL (dose adapted according to lean body weight and iodine concentration) of CM such as 370 mgI/mL iopromide (Ultravist, Bayer - Germany) or 350 mgI/mL iomeprol (Iomeron, Bracco - Italy) and should be carefully interpreted using focused small-FOV axial and sagittal viewing along the anatomical orientation of the vagina [23].

**Fig. 3:**
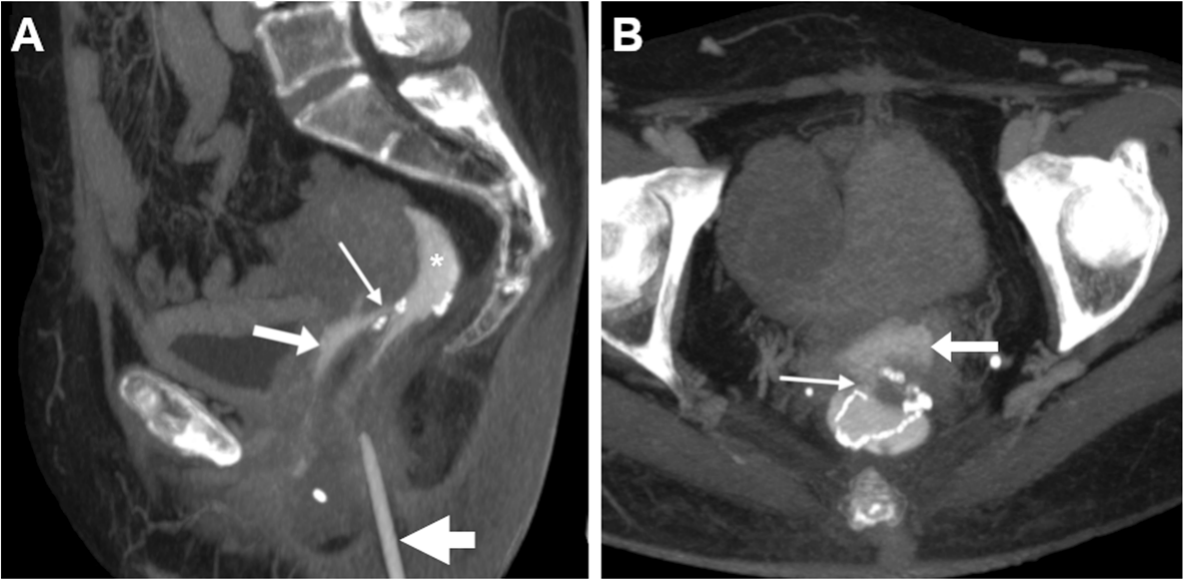
CT with retrograde contrast medium (CM) enema in VF following low anterior resection (LAR) for rectal carcinoma: maximum-intensity projection (MIP) CT reconstructions (**a**, **b**) show contrast medium flowing from the diverted stump into the vagina (arrows) through the patent VF (thin arrows). Note rectal catheter (thick arrow in **a**)

In selected patients with suspected recto- or colo-VF, CT may be repeated following administration of diluted CM such as diatrizoate meglumine (Gastrografin, Bayer - Germany) or 3–5% iomeprol (Iomeron, Bracco - Italy) via a rectal probe. Borrowing from experience with investigation of leaking colorectal surgical anastomoses, CM enema (Fig. 3) complemented with maximum-intensity projection (MIP) reconstructions may be beneficial to provide the definitive confirmation of VF and directly visualise visualise the abnormal communication that leads to vaginal opacification [24].

### CT indications and techniques for the lower urogenital tract

Nowadays, CT-urography represents the preferred technique to obtain a comprehensive evaluation of the urinary tract and is generally warranted in patients with suspected iatrogenic injuries [25]. A variety of strategies exist for improving the ensuring adequate patient hydration and improving opacification of the collecting systems, ureters and bladder [26].

Shortly after surgery, a preliminary unenhanced acquisition is helpful to detect post-operative fluid collections such as urinomas and hyperattenuating fresh bloozd at the vaginal vault, in the pelvis and peritoneal cavity. Classic CT-urography protocols include corticomedullary, nephrographic and excretory acquisitions, obtained 30 s, 90–100 s and 8–10 min after start of CM injection, respectively. Split-bolus CT techniques may be beneficial in the setting of suspected iatrogenic injury, as they provide a single acquisition that combines adequate opacification and distension of the urinary tract with either nephrographic [27] or corticomedullary/vascular plus nephrographic [28] phases of enhancement, thus limiting the ionising radiation dose. CT-urography studies benefit from direct supervision by the attending radiologist for planning and to assess the need for additional acquisitions. In our experience, moving the patient from the supine to the prone position and ultra-delayed acquisitions (20 to 60 min) are frequently helpful to assess or confidently exclude urine extravasation. The routine reconstruction of small-FOV sagittal and oblique images of the pelvis is beneficial to elucidate anatomy and abnormalities of the female genital organs [25, 26].

Unfortunately, opacified urine in the bladder at the end of CT-urography often does not suffice to open a VF by pressure. Therefore, in patients with clinical suspicion of iatrogenic bladder injury or fistulisation, multidetector CT-cystography may be performed with a preliminary passive infusion of 8–10% diluted CM such as 370 mgI/mL iopromide (Ultravist, Bayer - Germany) or 350 mgI/mL iomeprol (Iomeron, Bracco - Italy) in normal saline through the Foley catheter, monitored by means of CT scanograms and continued until either the patient complains of intolerable distension, flow stops or the radiologist sees an adequately filled bladder. Both excretory-phase CT-urography and CT-cystography studies should be reviewed on multiple planes using CT-angiography window setting (width 600–900 HU, level 150–300 HU) and performing MIP reconstructions, to detect or confidently exclude opacified urine leaks and fistulas [25, 29].

In patients with suspected VF, a vaginal tampon may be placed before performing either CT-urography or CT-cystography. In preliminary unenhanced images, tampons act as negative contrast and allow better identification of the vagina and uterine cervix. After urinary opacification, tampons may soak with CM and become hyperattenuating if a VF is present [30]. Alternatively, other Authors suggest preliminary vaginal filling using 100 to 180 mL of ultrasound gel before CT-cystography [31].

**Fig. 4:**
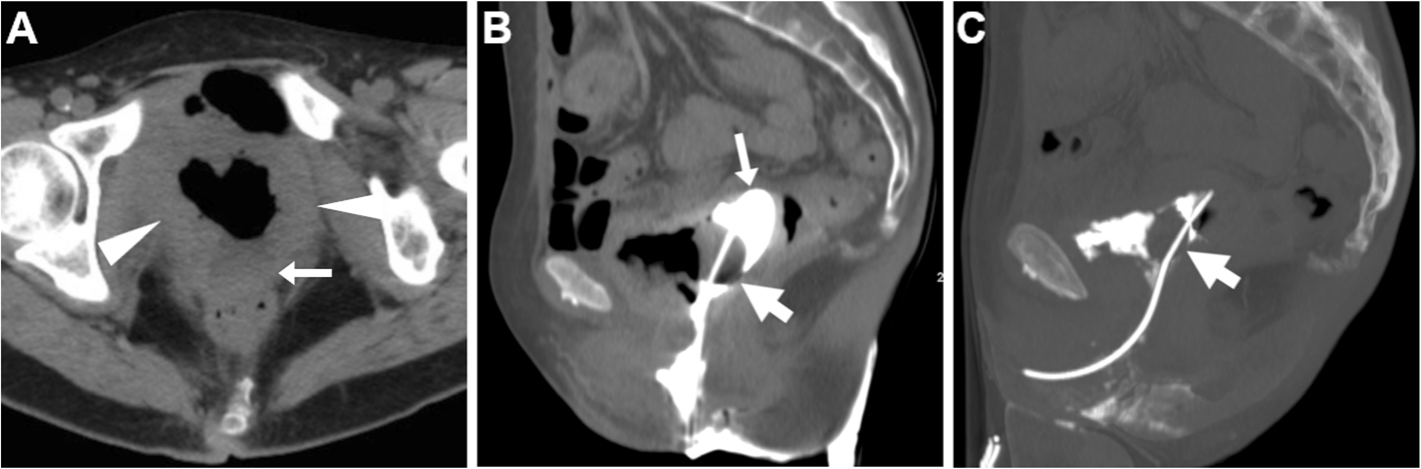
CT-vaginography in a patient with advanced bladder and urethral undifferentiated carcinoma invading the vagina, seen as marked circumferential mural thickening (arrowheads in precontrast CT **a**). After introduction and inflation of the Foley catheter (thick arrows) in the vagina, injection of CM medium allows opacification and distension of the vagina (arrow at vaginal dome in **b**) and subsequent CM flow anteriorly into the urinary bladder (in **c**)

Finally, CT-vaginography (Fig. 4) combines the cross-sectional anatomy with functional information concerning patency of the VF, but is cumbersome as it requires inflation of a Foley catheter into the vagina followed by injection of CM [32].

## Cross-sectional imaging appearances of entero-vaginal fistulas

### Indirect CT and MRI signs

At MRI, the normal vagina is a collapsed fibromuscular structure situated between the urinary bladder and the rectum with an H-shaped configuration in axial sections and elongated J-shape in sagittal views [17, 18].

**Fig. 5:**
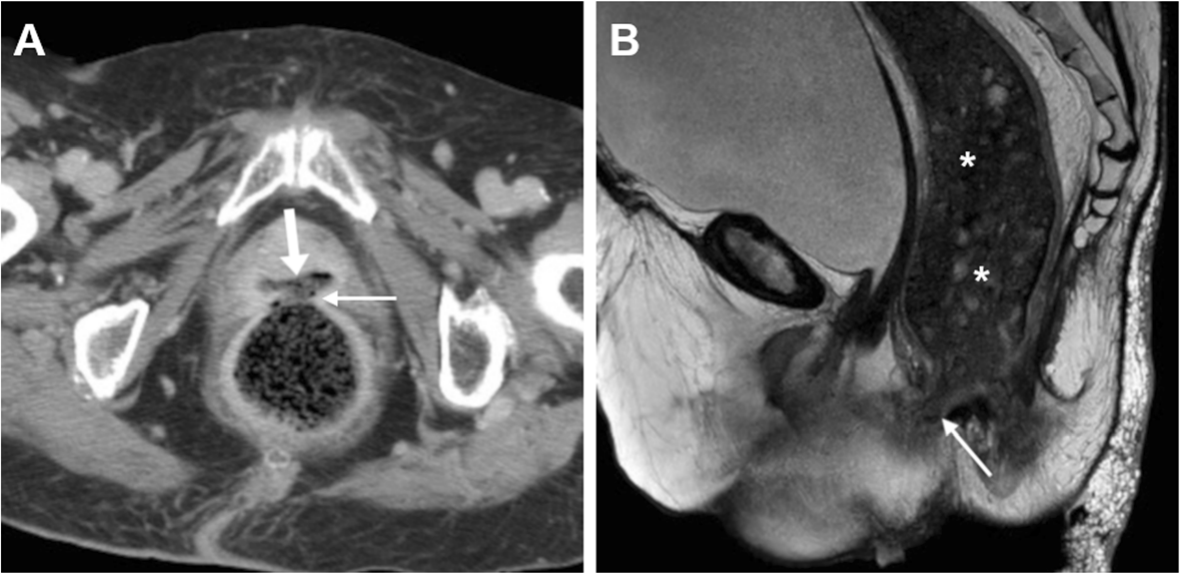
Two different radiation-induced recto-VF in elderly women with history of pelvic radiotherapy. **a** Axial CT image shows midline fistula (thin arrow) filled by faeces connecting the anterior aspect of the rectum to the vagina (arrow). **b** Sagittal T2-weighted MRI image shows a 1-cm wide fistula (thin arrows) between the most distal aspect of stool-filled rectum (*) and vagina

**Fig. 6:**
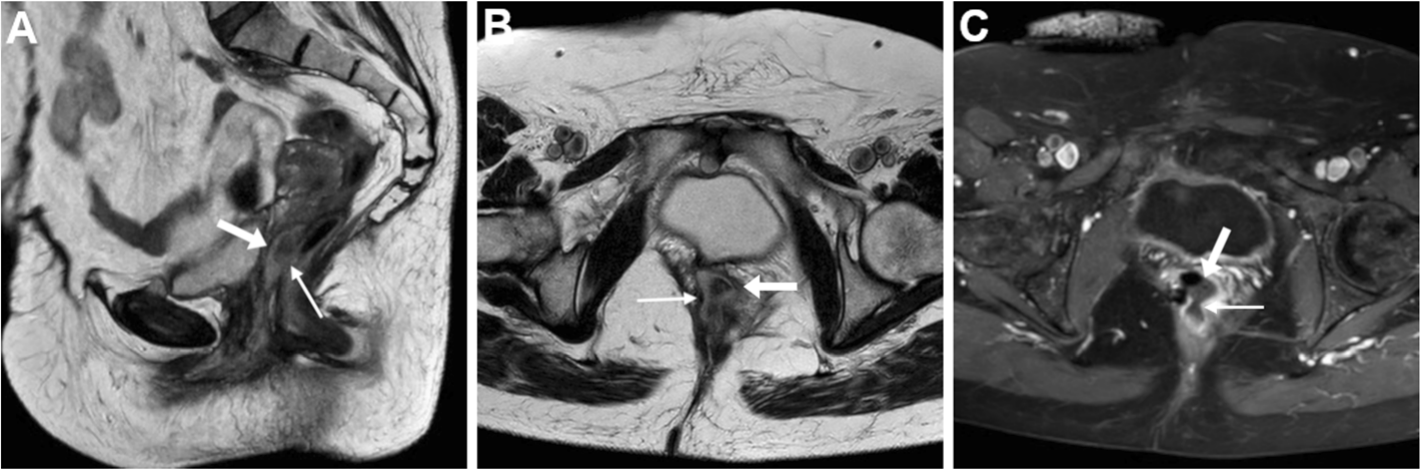
Recto-VF after surgical treatment of bladder and uterine prolapse with prosthetic mesh placement. T2-weighted (**a**, **b**) and FS post-gadolinium T1-weighted (**c**) MRI images show wide fluid-filled communication (thin arrows) between the distal rectum and upper third of the vagina (arrows), with marked peripheral enhancement in (**c**). Note poor visualisation of the mesh (few artefacts abutting the right side of the VF on **c**)

**Fig. 7:**
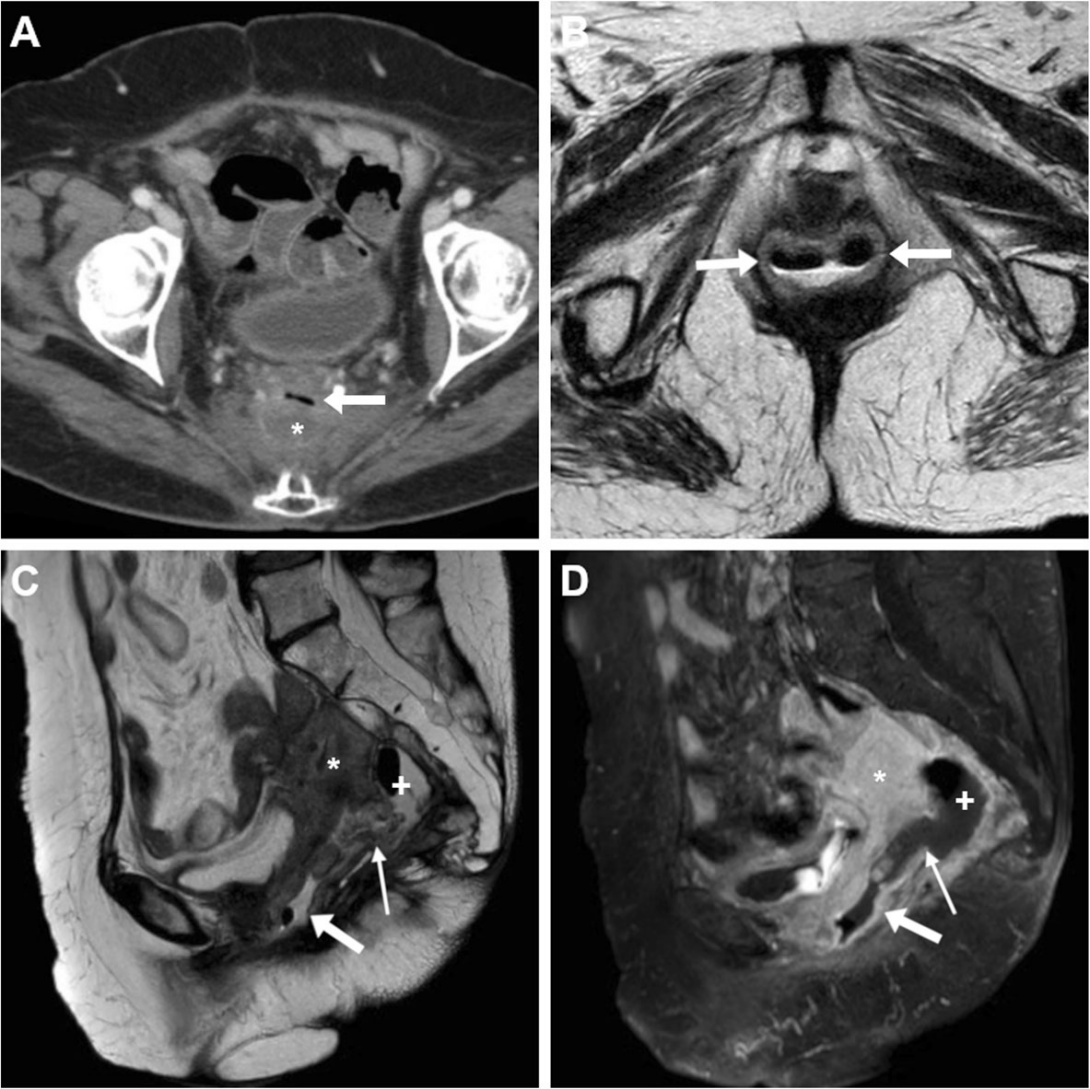
Neoplastic recto-VF, CT and MRI findings of VF-related vaginitis. In a 72-year-old with previous hysterectomy and resected rectal carcinoma, CT (**a**) shows local recurrence (*) indissociable from the air-filled vagina (arrows). Despite impossible gynaecological exploration, T2-weighted images (**b**, **c**) and fat-suppressed (FS) post-gadolinium T1-weighted (**d**) MRI images show increased solid tumour mass (*), air- and fluid-filled vagina (arrows) with circumferential wall thickening, oedematous T2 signal intensity and positive enhancement. The fluid-filled communication (thin arrows) between presacral air-fluid collection (+) and vagina is consistent with VF

At both CT and MRI, the identification of abnormal vaginal distension by intraluminal stool (Figs. 5 and 6) or air (Fig. 7) should alert the radiologist to suggest the presence of an entero-VF Alternatively, a distended vagina with intraluminal simple fluid is seen in both entero- and urinary VF [6, 17, 18].

**Fig. 8:**
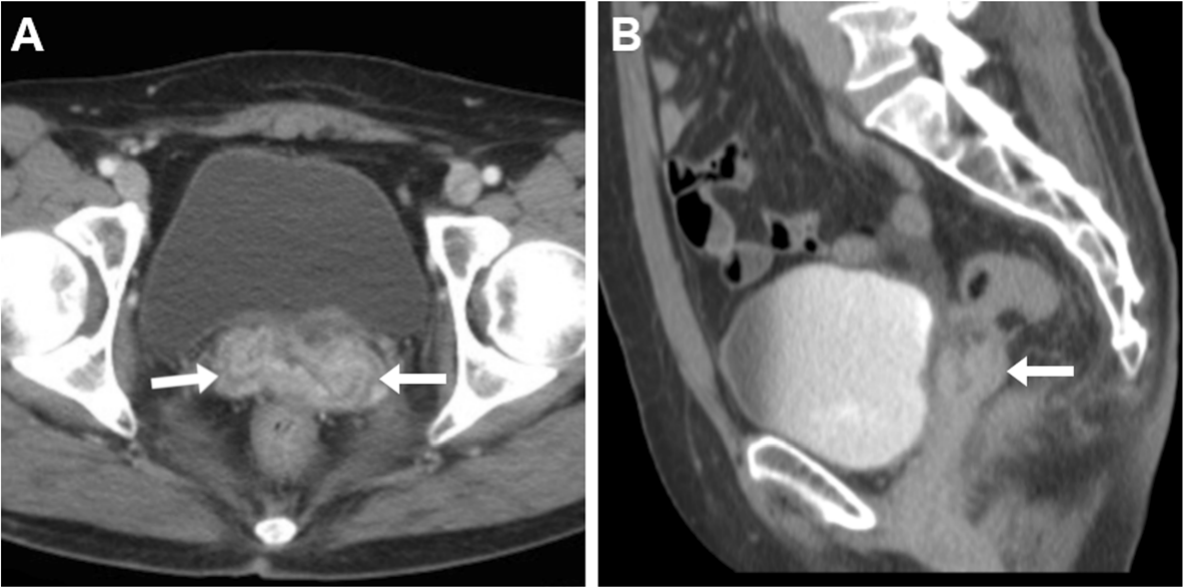
Differential diagnosis of VF-related vaginitis: expected CM-enhanced CT appearance in axial (**a**) and sagittal (**b**) planes after recent vaginal hysterectomy with a “swollen” configuration in transverse planes, circumferential oedematous mural thickening and marked mucosal enhancement

Furthermore, MRI may well depict the associated inflammation of the vagina, seen as diffuse mural thickening with oedematous hypersignal, obliteration of the normally T2-hypointense submucosa layer and prominent CM enhancement (Fig. 7) [6, 17, 18]. Alternatively to a VF, similar cross-sectional inflammatory changes may result from infectious vaginitis from various pathogens, albeit this condition rarely requires directed imaging. Furthermore, a similar appearance that reflects diffuse vaginal oedema is normally observed after recent vaginal hysterectomy (Fig. 8) [25].

### Direct CT and MRI signs

The cross-sectional imaging hallmark of an entero-VF is represented by a variably oriented track that interconnects the vagina with the sigmoid colon, rectum or anus. Radiologists should focus on which part of the vagina is affected, since this information is particularly relevant for choosing the appropriate surgical approach. Broadly, in sagittal viewing at both CT and MRI, vagina can be subdivided in upper (at the level of the lateral vaginal fornices), middle (at the level of the urinary bladder base) and lower third (at the level of the urethra) [2, 6].

**Fig. 9:**
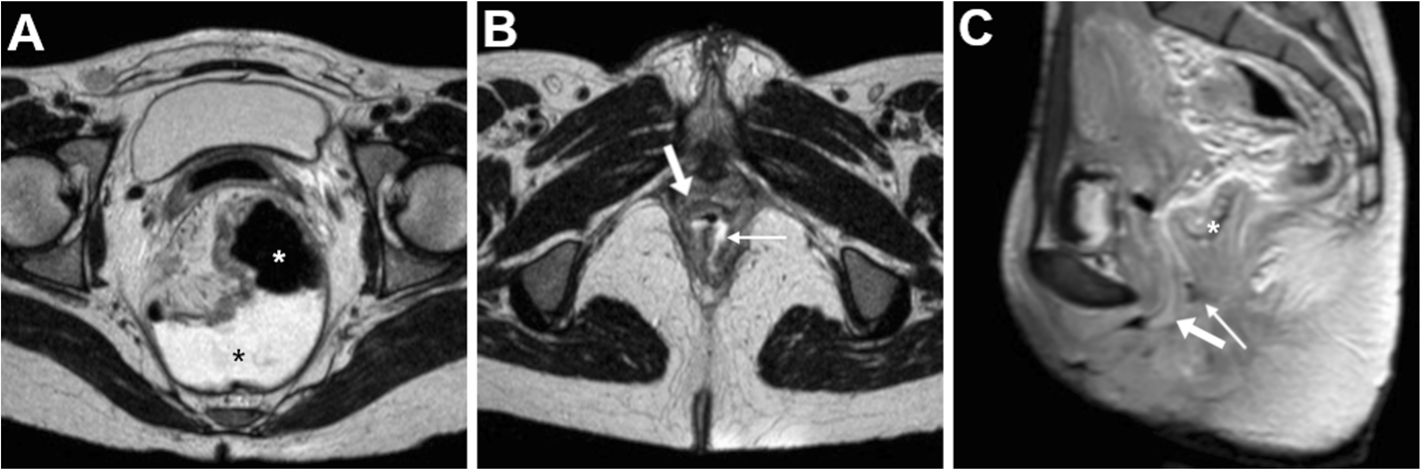
Ano-VF following total proctocolectomy with ileal pouch-anal anastomosis (IPAA) in a 44-year-old with ulcerative colitis. Axial T2-weighted (**a**, **b**) and sagittal post-gadolinium T1-weighted (**c**) MRI images show fluid distension of the ileal pouch (*) and a small midline fistula (thin arrows) connecting the IPAA to the vagina (arrow)

**Fig. 10:**
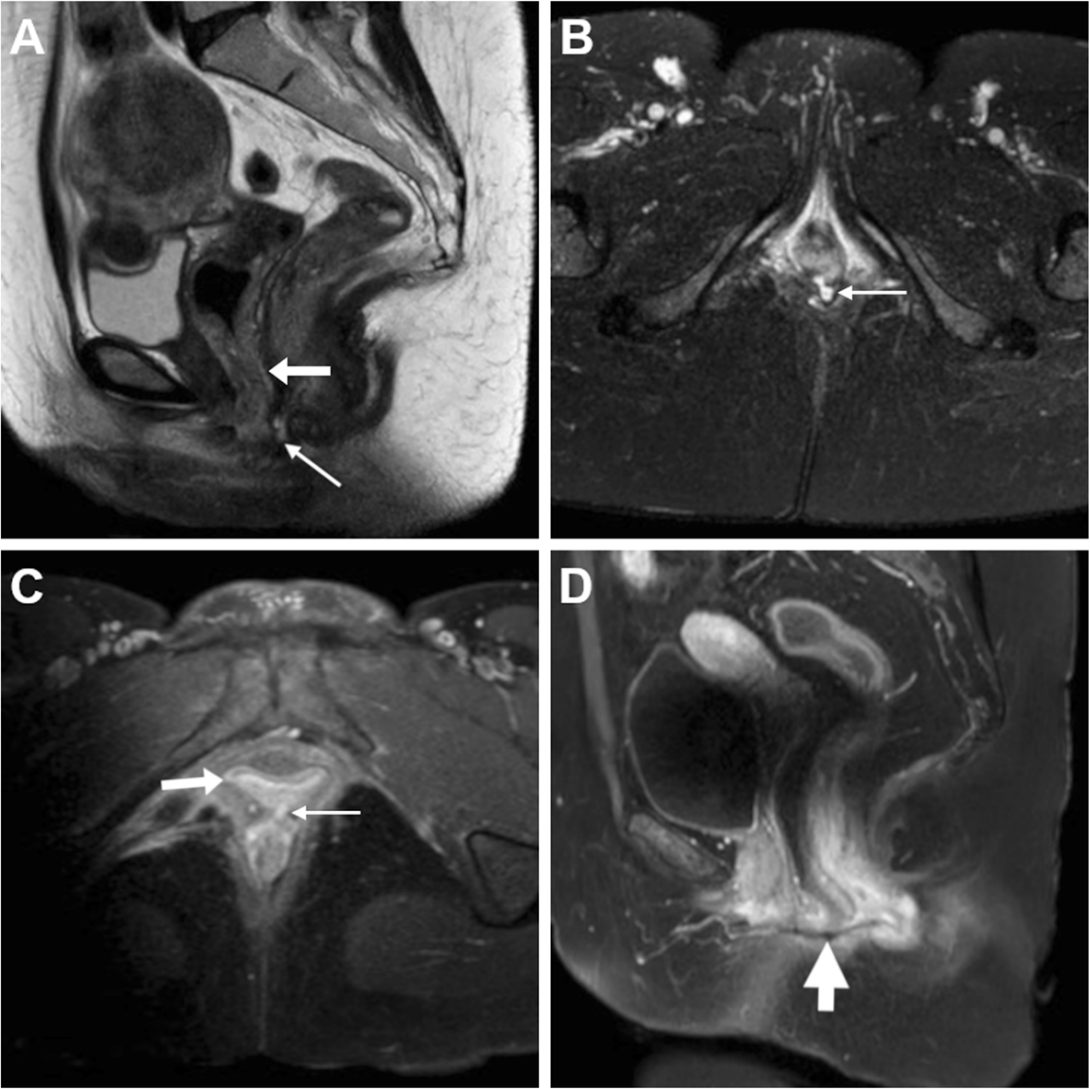
MRI of uncomplicated perianal Crohn’s disease (CD). **a**, **b** in a 41-year-old, the ano-VF is seen as a midline fluid-filled track (thin arrows) coursing through the anovaginal septum to reach the lower third of the vagina (arrow) on sagittal (**a**) and FS axial (**b**) T2-weighted images. **c**, **d** in a 36-year-old with colonic CD, the ano-VF is initially seen as a thin midline tract (thin arrow) on axial FS T1-weighted image after gadolinium CM (**c**) that reaches the vagina (arrow). On infliximab, repeated MRI including sagittal FS T1-weighted image after gadolinium CM (**d**) shows positioning of a seton (thick arrow) through the ano-VF

**Fig. 11:**
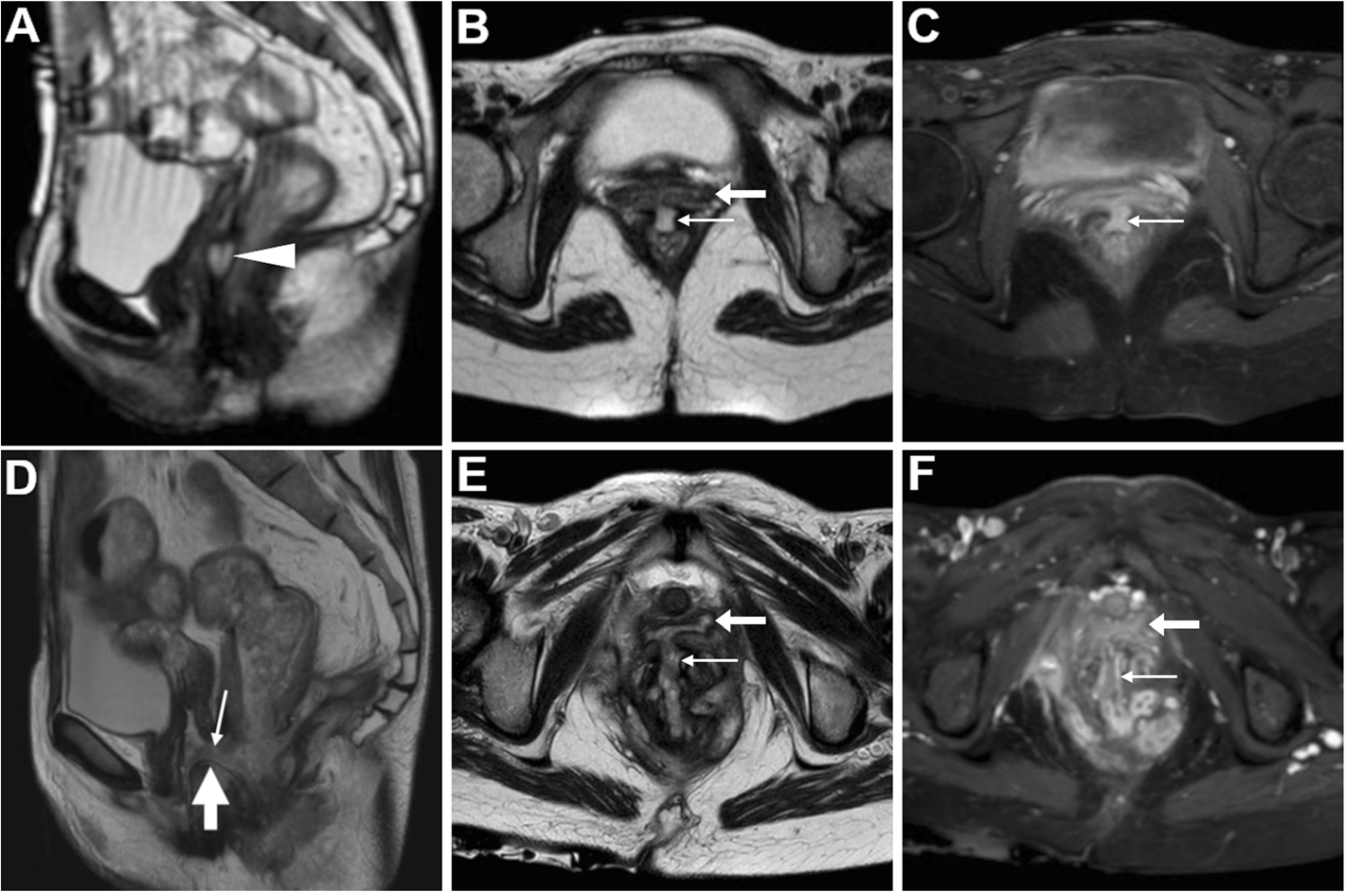
MRI follow-up of worsening perianal inflammatory disease in a 40-year-old with severe, refractory CD. Initial MRI (**a**-**c**) shows a small-sized abscess of the anovaginal septum (arrowhead in **a**) originating from a midline recto-VF (thin arrows) with strong enhancement on FS T1-weighted image after gadolinium CM (**c**). Two years later, despite positioning of a seton (thick arrow in **d**), repeated MRI (**d**-**f**) shows development of complex perianal disease including widened recto-VF (thin arrows) and formation of abscesses in both levator ani muscles. Note vagina (arrows)

The entero-VF may be stool- (Figs. 5 and 6), air- or fluid-filled (Figs. 9, 10 and 11). At MRI, active ano- and recto-VF generally appear as short tubular structures with high T2-weighted signal intensity reflecting a combination of intraluminal fluid and oedematous walls. Compared with the T1-hypointense fluid content, after CM administration, VF margins show intense inflammatory enhancement corresponding to the inflamed granulation tissue (Figs. 6, 7, 9 and 10). Conversely, chronic fistulous tracks show scar-like low T2-weighted signal and usually do not enhance [33]. When present, associated abscesses appear as fluid-filled cavities with internal near-water CT hypoattenuation, high T2-weighted MRI signal intensity and restricted diffusion reflecting the presence of pus, demarcated by an intensely enhancing peripheral wall (Fig. 11) [6, 17, 18].

### Colovaginal fistulas

**Fig. 12:**
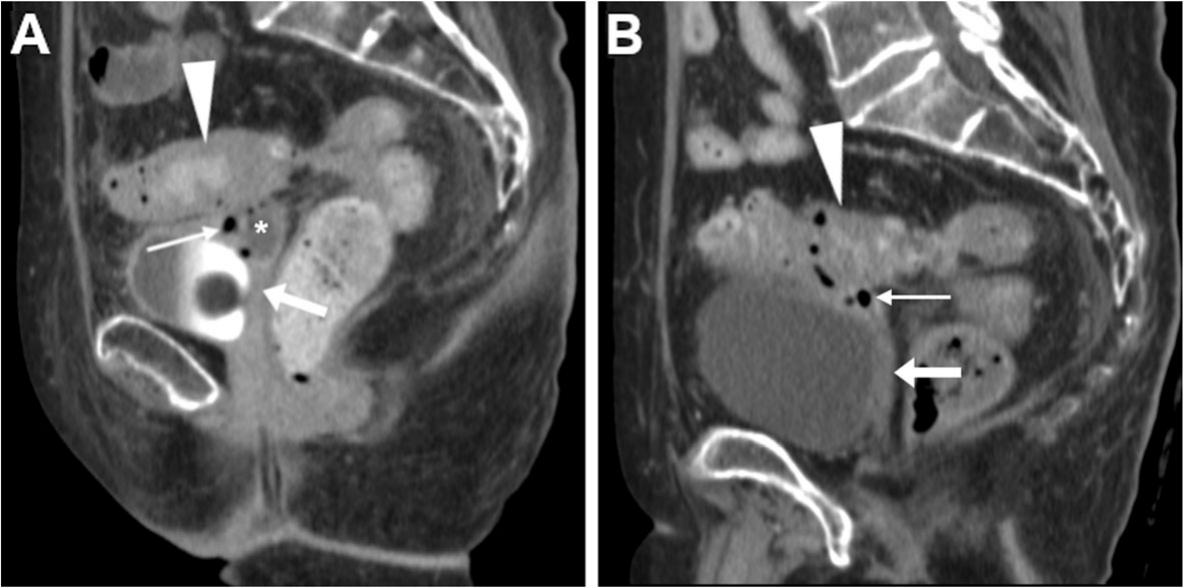
Surgically confirmed colo-VF in a 76-year-old with history of hysterectomy. During acute diverticulitis and *Clostridium difficile* colitis, CT (**a**) shows a small-sized abscess (*) at the vaginal dome (arrow), closely abutting the thickened walls of the sigmoid colon (arrowhead), which resolves after conservative treatment (**b**). Note air bubbles (thin arrows) in the vaginal stump

The typical diverticulitis-related colo-VF communicate between the inferior aspect of the sigmoid colon to the midline or left lateral aspect of the vaginal dome, and is identified in approximately 60% of cases as a short vertical tract on coronal and sagittal CT images. An abscess cavity may be interposed between the two structures (Fig. 12a) [34, 35].

In patients with consistent clinical findings, the detection of adhesion between the thickened wall of the sigmoid colon and the vaginal dome and the associated presence of air into the vaginal lumen (Fig. 12) are sufficient to confirm the diagnosis. Alternatively, the same findings should be reported as suspicious of VF and warrants focused gynaecological examination [2, 6, 7].

Surgical repair of colo- and high recto-VF is performed via abdominal approach to remove the diseased colonic segment, and frequently requires a temporary diverting stoma and interposition of omental or epiploic fat [2, 11].

### Rectovaginal fistulas

Typically anteriorly oriented and located on or near the midline, recto-VF are recognizable on sagittal CT and MRI images and connect the posterior aspect of vagina and anterior wall of rectum (Figs. 5 and 6). Compared with colo- and ano-VF, recto-VF are often wider and associated with identifiable discontinuity or cleft in the facing vaginal and rectal walls. In patients with post-surgical VF, CT with optional contrast enema (Fig. 3) represents the preferred technique and the anastomotic site is readily identified by the presence of surgical staples. Whereas CT clearly shows metal, on MRI (Fig. 6), the presence of suture materials is suggested by susceptibility artefacts, more prominent on gradient-echo T2-weighted images [2, 6, 7].

The detection of solid pelvic tissue is the hallmark of a neoplastic aetiology of the recto-VF (Fig. 7) [2, 6, 7, 36].

Depending on fistula site, cause and features, surgical management may be performed via transabdominal, transperineal, endorectal or vaginal (in low recto- and ano-VF) approach. Excision may be completed with interposition of autologous tissue (such as Martius flap or gracilis muscle). If present, prosthetic mesh should be removed. Alternatively, biomaterials such as fistula plugs or fibrin adhesive glues may be positioned [11, 37].

### Anovaginal fistulas

Ano-VF depart at or below the dentate line and are often challenging to diagnose at imaging, particularly in lean women with very thin adipose tissue between the vagina and anus. The characteristic MRI appearance is a short midline or paramedian T2-hyperintense and enhancing band, best seen on FS sequences, which cross anteriorly the thin anovaginal septum (Figs. 9 and 10). Radiologists should report the site and direction of ano-VF described using the surgical “anal clock” scheme with the patient in the lithotomy position, in which the vagina is located between 11 and 1 o’clock and the natal cleft is at 6 o’clock position. Abscesses (Fig. 11) and signs of vaginal inflammation may coexist. In our experience in CD patients, perianal MRI has adequate accuracy (over 90%) for ano-VF, despite the fact that they are often short, small and collapsed [20, 21, 38–40].

Although explanation of the anatomical Parks’ [41] and St. James’ hospital [42] classifications lies beyond the scope of this article, MRI allows classification of perianal inflammatory disease according to the relationships with the internal and external sphincter muscles and therefore differentiation between inter-, trans-, supra- and extra-sphincteric fistulas; therefore, MRI contributes to the distinction between simple or complex perianal disease according to the American Gastroenterological Association (AGA) criteria [20, 21].

**Fig. 13:**
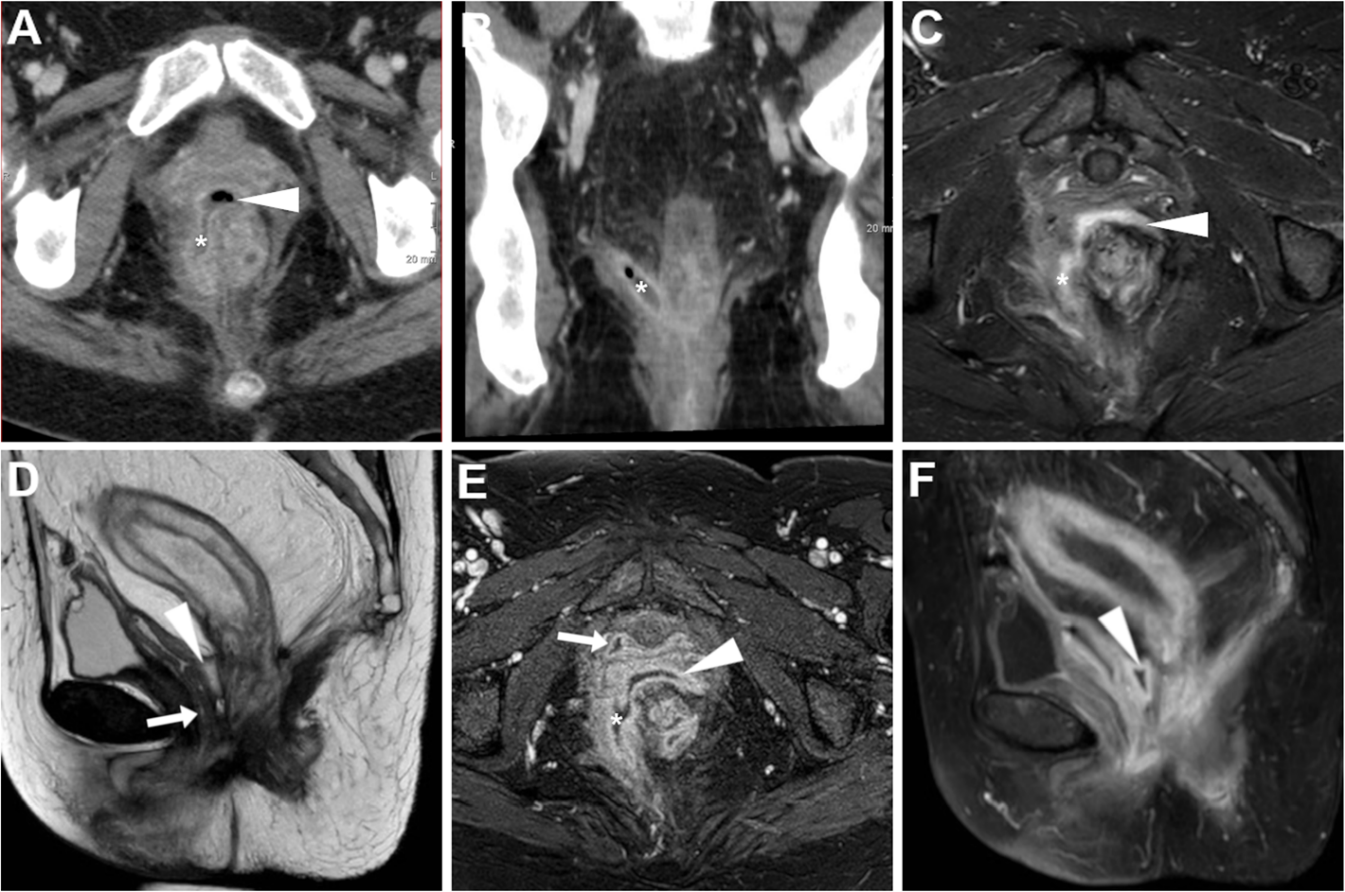
MRI-like use of CT to investigate CD-related perianal inflammatory disease. In a 50-year-old, small-FOV axial (**a**) and oblique-coronal (**b**) CT reformatted images show peripherally enhancing abscess collection extending from the anovaginal septum (arrowheads) to the right levator ani muscle (*). Axial FS (**c**) and sagittal (**d**) T2-weighted, post-gadolinium FS axial (**e**) and sagittal (**f**) T1-weighted MRI images show same findings with fluid-filled abscess cavities with strong mural enhancement. Note severe proctitis as circumferential thickening of rectal walls with oedematous submucosa (in **d**) and marked homogeneous enhancement (in **f**)

Patients with CD tend to have complex perianal disease including wide ano-VF, multiple or branching fistulas, intersphincteric “horseshoe” and levator ani abscesses (Figs. 11 and 13), and generally warrant MRI to detect abscesses and guide surgical examination under anaesthesia before starting anti-TNFα therapies such as infliximab. Furthermore, MRI provides a consistent follow-up following surgery and seton placement (Figs. 10d and 11d) [39, 40].

## Cross-sectional imaging appearances of urinary vaginal fistulas

### Direct CT and MRI signs

**Fig. 14:**
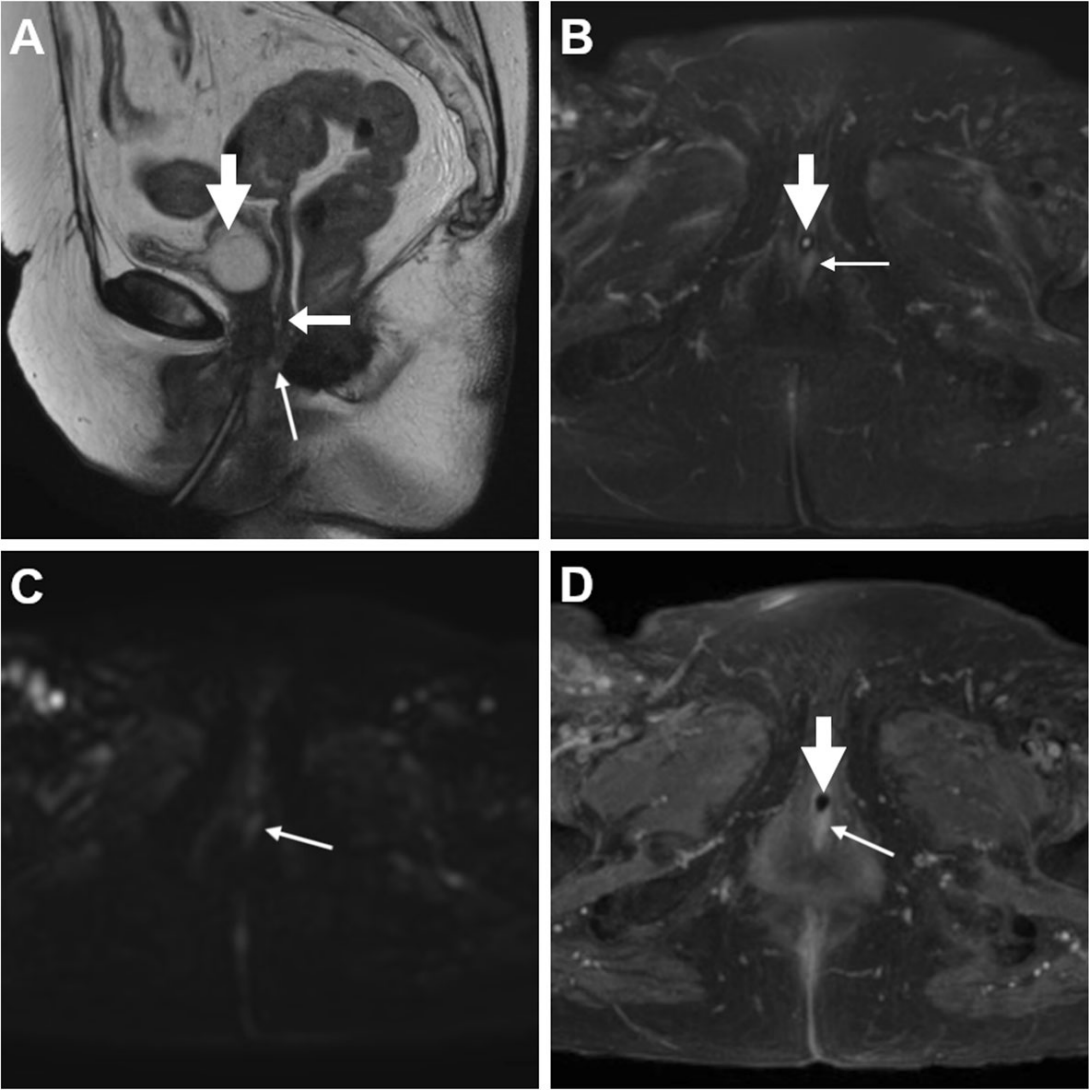
Urethro-VF in an elderly woman seen at MRI as subtle midline track (thin arrows) between the distal third of the vagina (arrow) and urethra containing Foley catheter (thick arrows), with high signal intensity on T2- (**a**), FS T2-weighted (**b**) and high *b* value DW (**c**) sequences, positive enhancement on post-gadolinium FS T1-weighted acquisition (**d**)

On targeted MRI, urethro-VF may be identified as more or less subtle fistulous tracks similar to ano-VF that course between the anterior aspect of the vagina and the target-like female urethra (Fig. 14) [43, 44].

**Fig. 15:**
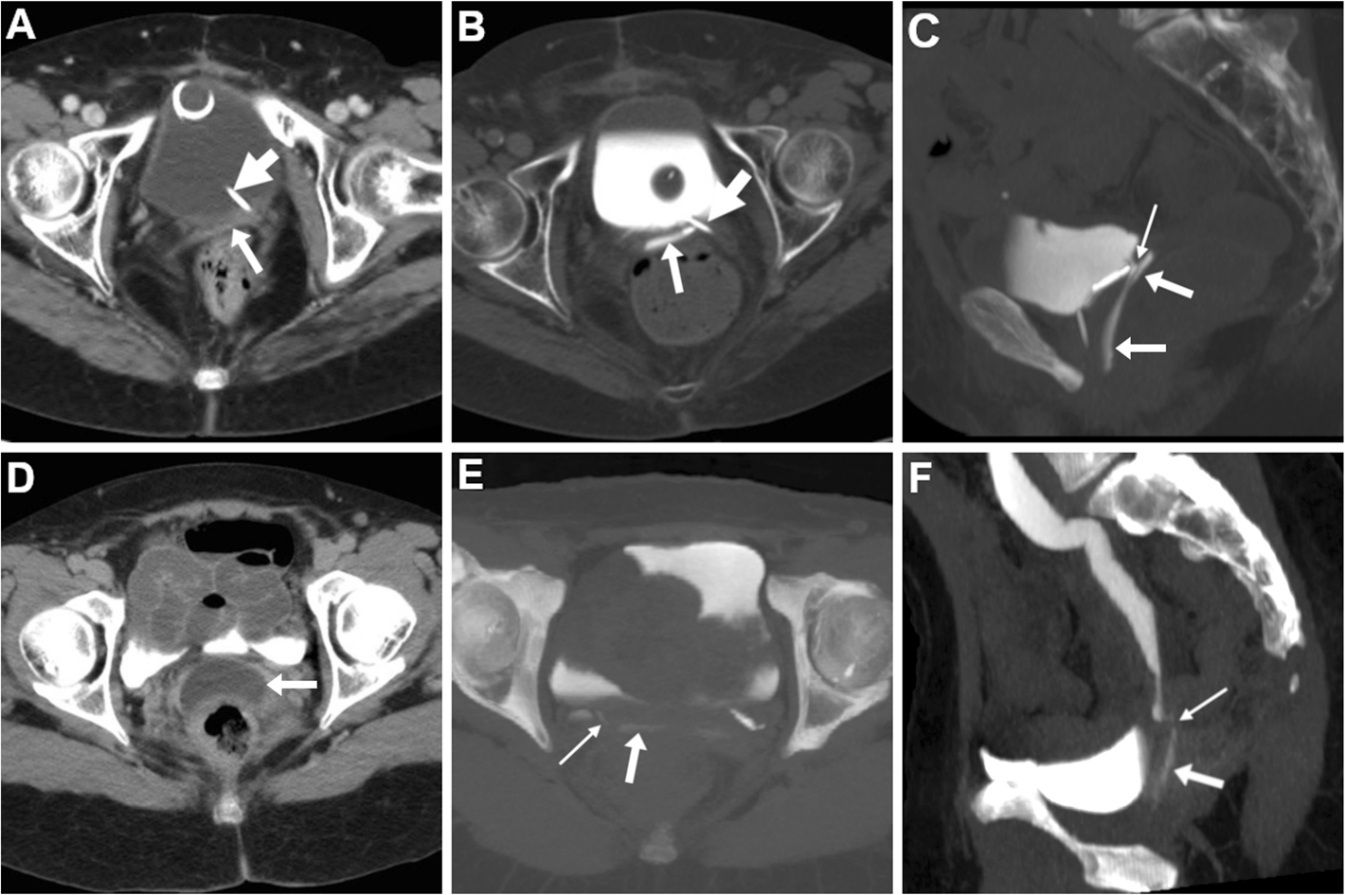
Two cases of iatrogenic urinary VF. **a**–**c** After recent surgery for recurrent endometrial carcinoma, CT-urography (note ureteral stent indicated by thick arrows) shows left lateral retraction and opacification of the vagina (arrows) through a short vesico-VF (thin arrow in **c**) [Adapted from Open Access Ref. no. [25]]. **d**–**f** Following radical hysteroannessectomy for endometrial carcinoma, CT-urography shows marked fluid-filled dilatation of the vagina (arrow in **d**). On ultra-delayed (30 min) acquisition (**e**, **f**), some opacified urine flows into the vagina (arrows) through a leaking uretero-VF (thin arrows)

**Fig. 16:**
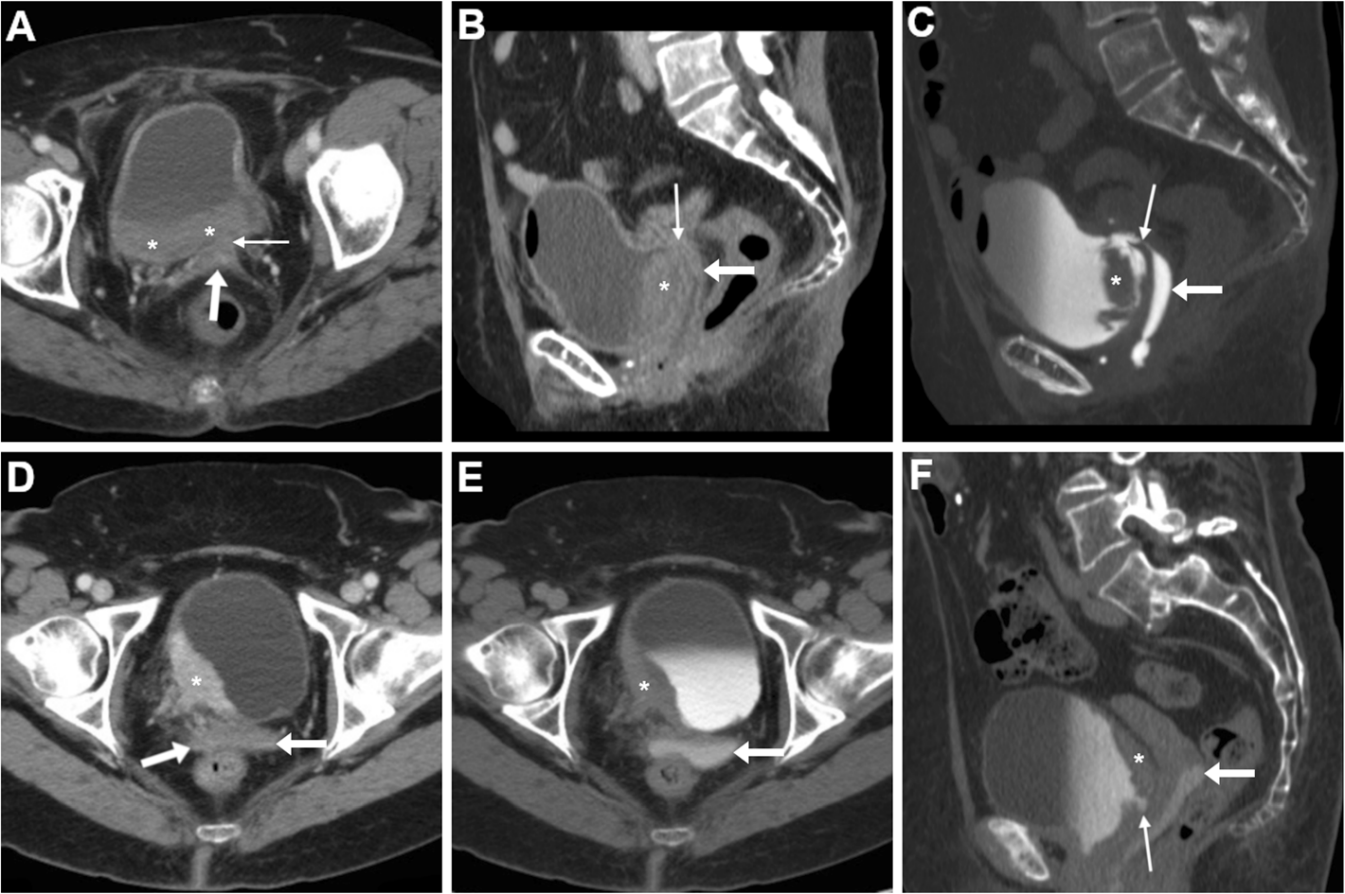
Two cases of urinary VF complicating bladder cancers. **a**–**c** In a patient with previous hysterectomy, CT (**a**, **b**) shows extensive mural thickening (*) of the posterior bladder aspect, causing adhesion and focal retraction of the bladder dome, and corresponding filling defect on excretory phase acquisition (**c**). The patent VF (thin arrows) leads to opacification of the vagina (arrows). **d**–**f** In a patient with strongly enhancing tumour (*) of the right posterolateral aspect of urinary bladder (**d**), excretory phase acquisition (**e**, **f**) shows vaginal opacification (arrows) via a short VF (thin arrow in **f**)

On either CT-cystography or excretory-phase CT-urography, a urinary VF is heralded by the presence of opacified urine in the vagina (Figs. 15 and 16). Uretero- and vesico-VF are identified as urine-filled tracks that connect the vagina to the distal ureter (Fig. 15) or bladder (Fig. 16), respectively [25, 45, 46]. Alternatively from post-surgical ones, VF may result from locally advanced tumours of the urinary bladder and/or female urethra, which are recognised as abnormal solid, enhancing mural thickening that infiltrates the vagina (Figs. 4 and 16) [36].

Small non-malignant urethro- and vesico-VF may be managed conservatively with prolonged catheterisation or undergo electrocoagulation. Transvaginal surgical repair with or without flap techniques may be either performed early or postponed after healing of inflammation and necrosis, aiming to reduce morbidity. Similarly to iatrogenic and traumatic bladder rupture, a transabdominal approach is required in complex injuries, intraperitoneal leaks and cranial vesico-VF [12, 47, 48].

### Differential diagnosis

Sinus tracts differ from VF in that they do not connect to an organ but are blind-ending or terminate into an abscess collection [20, 21].

**Fig. 17:**
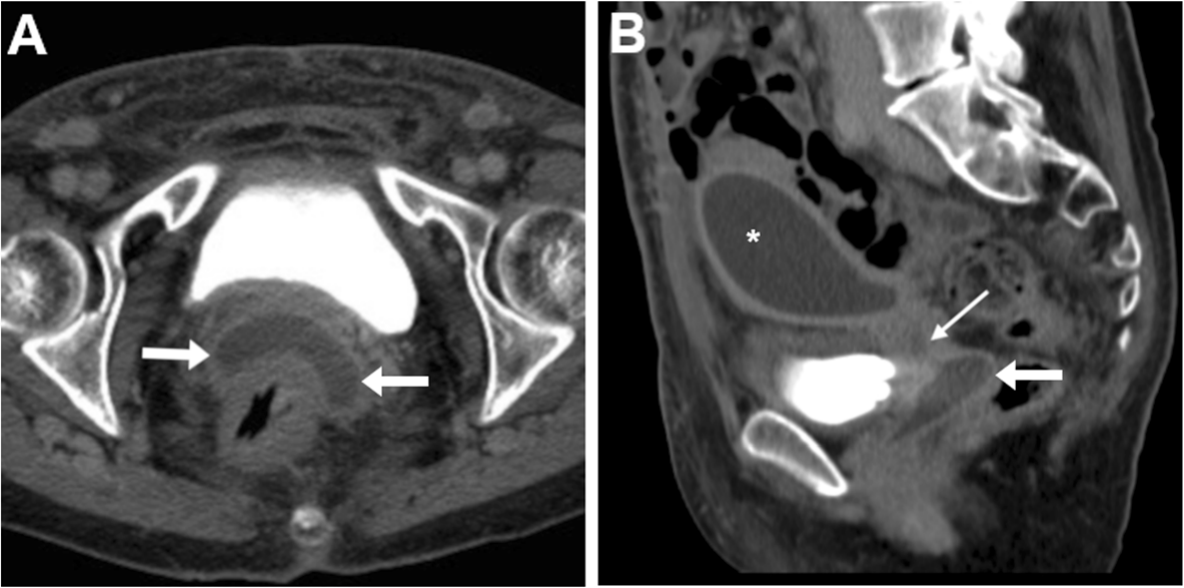
Differential diagnosis: rare case of lymphatic VF in a 67-year-old with previous laparotomic surgery for advanced ovarian cancer. CT (**a**, **b**) shows fluid-filled dilatation of the vagina (arrows) communicating via a fistula (thin arrow in **b**) with an ovoid fluid-attenuation pelvic collection (* in **b**) consistent with postoperative lymphocele

Rare conditions that may mimic a VF both clinically and at imaging are peritoneal and lymphatic fistulas (Fig. 17), in which communication is established between the vaginal dome and a post-operative fluid or lymph collection. Differentiation relies on correct identification of the abnormal collection that does not fill with enhanced urine [49].

## Conclusion

In the developing world, obstructed labour and perineal lacerations during spontaneous or instrumental delivery remain the most prevalent causes; conversely, in Western countries, VF are increasingly iatrogenic in nature as they develop as complications of various pelvic, urologic and gynaecologic procedures. Although uncommon, VF result in substantial morbidity for patients. The ideal cross-sectional techniques depend on the anatomic site and affected organ. With appropriate acquisition and focused interpretation, state-of-the art CT and MRI provide optimal visualisation of entero- and urinary VF that is crucial for correct therapeutic choice and surgical planning.

## Abbreviations

AGA: American Gastroenterological Association
CD: Crohn’s disease
CM: Contrast medium
CT: Computed tomography
DW: Diffusion-weighted
ECCO: European Crohn’s and Colitis Organisation
FOV: Field-of-view
FS: Fat-suppressed
HU: Hounsfield units
IPAA: Ileal pouch-anal anastomosis
LAR: Low anterior resection
MIP: Maximum-intensity projection
MRI: Magnetic resonance imaging
VF: Vaginal fistula
